# Establishment of a deep-learning-assisted recurrent nasopharyngeal carcinoma detecting simultaneous tactic (DARNDEST) with high cost-effectiveness based on magnetic resonance images: a multicenter study in an endemic area

**DOI:** 10.1186/s40644-025-00853-5

**Published:** 2025-03-24

**Authors:** Yishu Deng, Yingying Huang, Haijun Wu, Dongxia He, Wenze Qiu, Bingzhong Jing, Xing Lv, Weixiong Xia, Bin Li, Ying Sun, Chaofeng Li, Chuanmiao Xie, Liangru Ke

**Affiliations:** 1 School of Electronics and Information Technology, Sun Yat-sen University, Guangzhou, 510006 Guangdong China; 2 State Key Laboratory of Oncology in South China, Guangdong Key Laboratory of Nasopharyngeal Carcinoma Diagnosis and Therapy, Guangdong Provincial Clinical Research Center for Cancer, Sun Yat-sen University Cancer Center, Guangzhou, 510060 China; 3 Department of Radiology, Sun Yat-Sen University Cancer Center, Guangzhou, 510060 China; 4 Information Technology Center, Sun Yat-Sen University Cancer Center, Guangzhou, 510060 China; 5 Department of Radiation Oncology, The First People’s Hospital of Foshan, Foshan, 528000 China; 6 Department of Radiation Oncology, Affiliated Cancer Hospital and Institute of Guangzhou Medical University, Guangzhou, 510095 China; 7 Department of Nasopharyngeal Carcinoma, Sun Yat-Sen University Cancer Center, Guangzhou, 510060 China; 8 Department of Radiation Oncology, Sun Yat-Sen University Cancer Center, Guangzhou, 510060 China; 9 Precision Medicine Center, Sun Yat-Sen University, Guangzhou, 510060 China

## Abstract

**Background:**

To investigate the feasibility of detecting local recurrent nasopharyngeal carcinoma (rNPC) using unenhanced magnetic resonance images (MRI) and optimize a layered management strategy for follow-up with a deep learning model.

**Methods:**

Deep learning models based on 3D DenseNet or ResNet frames using unique sequence (T1WI, T2WI, or T1WIC) or a combination of T1WI and T2WI sequences (T1_T2) were developed to detect local rNPC. A **d**eep-learning-**a**ssisted **r**ecurrent **N**PC **de**tecting **s**imultaneous **t**actic (DARNDEST) utilized DenseNet was optimized by superimposing the T1WIC model over the T1_T2 model in a specific population. Diagnostic efficacy (accuracy, sensitivity, specificity) and examination cost of a single MR scan were compared among the conventional method, T1_T2 model, and DARNDEST using McNemar’s Z test.

**Results:**

No significant differences in overall accuracy, sensitivity, and specificity were found between the T1WIC model and T1WI, T2WI, or T1_T2 models in both test sets (all *P* > 0.0167). The DARNDEST had higher accuracy and sensitivity but lower specificity than the T1_T2 model in both the internal (accuracy, 85.91% vs. 84.99%; sensitivity, 90.36% vs. 84.26%; specificity, 82.20% vs. 85.59%) and external (accuracy, 86.14% vs. 84.16%; sensitivity, 90.32% vs. 84.95%; specificity, 82.57% vs. 83.49%) test sets. The cost of a single MR examination using DARNDEST was $330,724 (internal) and $328,971 (external) with a hypothetical cohort of 1,000 patients, relative to $313,250 of the T1_T2 model and $340,865 of the conventional method.

**Conclusions:**

Detecting local rNPC using unenhanced MRI with deep learning is feasible and DARNDEST-driven follow-up management is efficient and economic.

**Supplementary Information:**

The online version contains supplementary material available at 10.1186/s40644-025-00853-5.

## Background

Nasopharyngeal carcinoma (NPC), a malignancy originating from the mucosal epithelium of the nasopharynx, is particularly prevalent in Southeast Asia and North Africa [1]. Despite advancements in radiotherapy and imaging techniques, treatment failure due to tumor recurrence remains a significant concern for NPC patients, with local recurrence occurring in 10% to 20% of cases in endemic regions [2–4]. Early detection and precise localization of recurrent NPC (rNPC) are essential for effective salvage treatment and improved prognosis [4–7].

However, detecting local rNPC poses challenges as it tends to grow submucosally, and deep-seated local rNPCs may be missed during clinical examination [8] or result in inaccessible endoscopic biopsy, thus thwart histopathological diagnosis. Hence, noninvasive and efficient modalities are urgently required for post-treatment assessment in NPC patients. Magnetic resonance imaging (MRI) has proven valuable in NPC management due to its excellent resolution in soft tissue, sensitivity in detecting bone marrow infiltration and advantage of multiparameter imaging [9], but its diagnostic efficacy in detecting local rNPC varies across studies, with sensitivity ranging from 56% to 83% and specificity from 78% to 83% [10, 11]. Radiation-induced anatomic distortion, alteration in signal intensity pattern, inflammatory reactions and scarring pose diagnostic difficulty in differentiating recurrent tumors from the variable appearance of radiation-induced alteration [12, 13].

Given the limitations of single imaging modalities, regular follow-up with repetitive MRI examinations is preferred for detecting local rNPC. However, repeated administration of MR contrast agent might increase the economic burden, mental stress and potential side effects for patients. Notably, MRI signals lack specific for distinguishing between recurrence and post-treatment changes, moreover, the 5-year local relapse-free survival has been increased from 66.6-90.8% to 87.7-95.5% in NPC patients with different stages of disease by introducing intensity modulated radiotherapy (IMRT) and comprehensive treatment strategy [14–16]. Deep learning algorithms have exhibited remarkable performance in lesion detection and segmentation by capturing intrinsic features of medical images. Deng YS et al demonstrated that contrast-enhanced magnetic resonance imaging (ceMRI) could be substituted in the identification and segmentation of NPC with the aid of deep learning models [17]. Likewise, the convolutional neural network-based algorithm could automatically discriminate between malignant and benign diseases at nasopharynx using T2-weighted fat-suppressed MR images [18]. Recently, MR-based deep learning models have enhanced radiologists' accuracy in detecting regional recurrent NPC [19]. This prompts the question of whether deep learning algorithms could aid in the differentiation between rNPC and radiation alterations, optimizing the follow-up strategy by selectively discarding contrast-enhanced MR examinations in certain subpopulation. This approach alleviates the economic burden on patients and national health systems and minimizes the potential risks associated with gadolinium-based contrast agents (GBCAs) in some extent.

To address that, deep learning models employed self-constrained 3D DenseNet or ResNet using unique sequence images (T1-weighted image [T1WI], T2-weighted image [T2WI], or post-contrast T1WI [T1WIC]) or their combination were developed to detect local rNPC. Consequently, we have demonstrated that unenhanced MRI is adequate in selective post-treatment NPC patients during follow-up with the aid of deep learning-based models and verified that in three different institutes. Moreover, an optimal **d**eep-learning-**a**ssisted **r**ecurrent **N**PC **de**tecting **s**imultaneous **t**actic (DARNDEST) based on MRI and a layer management strategy was developed, thereby providing an efficient, cost-effective, and lower-risk approach for NPC follow-up.

## Methods

### Patient enrollment

The participants in this study were previously subjects of research investigating the feasibility of detecting regional rNPC using deep learning-aided MRI [19]. In contrast, this research focused on local rNPC, with an emphasis on the viability of omitting enhanced MR examination and incorporating layer management for follow-up by introducing economic analysis.

**Fig. 1:**
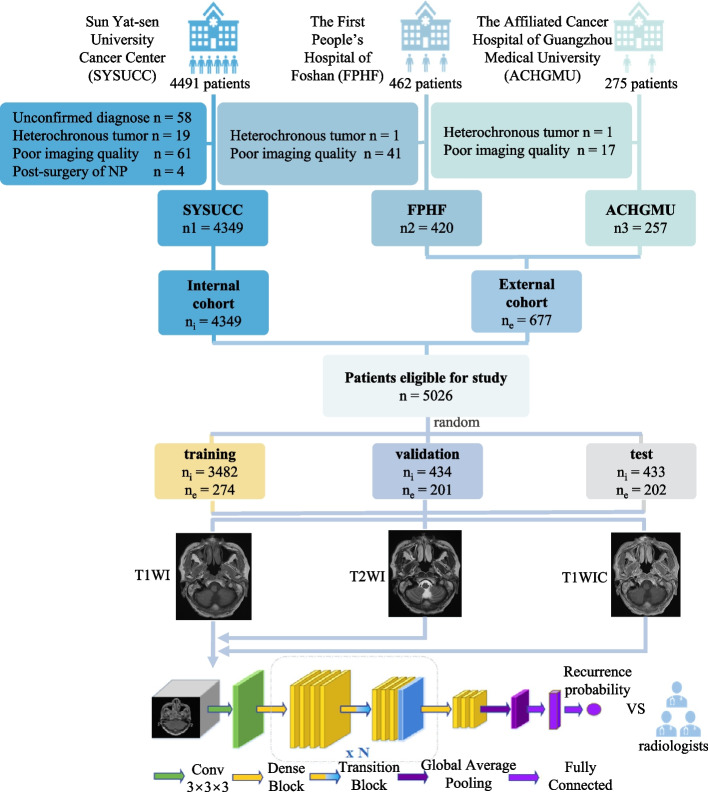
Flowchart of the participant enrollment and development of deep learning models

We searched the picture archiving and communication system (GE, USA) using the following terms and strategy: examination time = from Dec 01st, 2008 to July 31st, 2020, equipment = MR, examination type = plain and enhancement, region = head and neck, diagnosis = recurrent nasopharyngeal carcinoma or post-treatment or postradiotherapy alteration. The following cases were excluded: a) poor quality images due to artifacts or incomplete images; b) unconfirmed diagnosis; c) heterochronous tumor located at the nasopharynx or adjacent region; and d) surgery delivered to the nasopharynx. Finally, 4349, 420 and 257 eligible patients were enrolled from Sun Yat-sen University Cancer Center (SYSUCC), the First People’s Hospital of Foshan (FPHF) and the Affiliated Cancer Hospital of Guangzhou Medical University (ACHGMU), respectively. To enhance model robustness, external participants were combined into an "external cohort". The participants were randomly split into training, validation and test sets according to ratios of 8:1:1 and 1.4:1:1 in the internal and external cohorts, respectively (Fig. 1).

### Scanning parameters

Digital Imaging and Communications in Medicine images (DICOM) of T1-weighted (T1WI), and T2-weighted (T2WI) images preenhancement and T1-weighted images postenhancement (T1WIC) in the axial view were used for development, tuning and evaluation of the models. The images in the internal cohort were non-fat saturated, while those in the external cohort were fat saturated. Detailed information on the scanning machines and parameters was available in a previous study [19].

### Radiologists’ assessment and Image tagging

Initially, the image tagging was performed based on the imaging report. A junior radiologist (D.H.) with two years of experience, along with two radiation oncologists (H.W. and W.Q.) with over ten years of experience in head and neck malignancies, subsequently reviewed the image tagging in sequence and made necessary corrections according to the following criteria, 1) local recurrence referred to the detection of viable lesions in the nasopharynx or adjacent skull base/intracranial, including histopathology-confirmed nasopharyngeal carcinoma or those whose nasopharyngeal lesions increased in size as detected by time-serial images three months before or after the current MR examination, or who had suspicious nasopharyngeal lesions detected by the current MR examination that subsequently shrunk after anti-tumor treatment; 2) local recurrence-free referred to the absence of detectable viable lesions in the nasopharynx or adjacent skull base/intracranial on the current examination, with no evidence of recurrence, as defined above, after a follow-up period of one year. Notably, participants with regional recurrence, such as metastatic retropharyngeal or cervical lymph nodes, but no viable lesions in the nasopharynx or adjacent skull base/intracranial, were classified as negative for local recurrence. For cases lacking histopathological evidence and where there were different opinions between the junior radiologist and radiation oncologists, a senior radiologist (C.X.) with over thirty years of experience in head and neck malignancy made the final determination.

A senior radiologist (L.K.) with over ten years of experience in field of head and neck malignancy and a junior radiologist (Y.H.) with five years of experience participated in the test section. The two radiologists made diagnoses based on a single MR scan with complete DICOM images in the test set and were blinded to the histopathology and previous or subsequent examinations.

### Model development and tuning

Two commonly used deep learning model architectures, 3D DenseNet and 3D ResNet34 [20], were employed to investigate whether enhanced imaging could be replaced for the detection of local rNPC. PyTorch with 2 GPUs was utilized to train the 3D DenseNet and ResNet models for local rNPC detection using different sequences. To ensure the comparability of the performance of different models, we adopted the same hyperparameter settings and maintained the consistency of the training process. This includes using the same optimizer, loss function, minibatch size, learning rate adjustment strategy, data preprocessing, data augmentation methods, and model training monitoring. MR image preprocessing is described in supplementary methods. The detailed description of the 3D DenseNet model structure and a summary of its parameters are provided in the supplementary methods and supplementary Table 1, respectively. 'Adam' optimizer with binary cross-entropy loss, minibatch size 12, and initial learning rate 1e^-3^ was used for training. Data augmentation with RandomAffine [21] and an adaptive learning rate scheduling strategy was applied. Learning rate was reduced by half if validation set loss plateaued for 5 epochs and monitoring threshold set to 1e^-4^. Finally, models with best performance in validation set were chosen. The predicted values of T1WI and T2WI were combined with a weight of 0.5 to create T1_T2 model. The cutoff values for the predicted values of each model were determined using the Youden index (Supplementary Table 2).

### Optimization of the DARNDEST

Given the superior overall performance of the DenseNet model in detecting local rNPC compared to the ResNet model, the optimization of DARNDEST focused on the DenseNet model. To further improve the sensitivity of detecting local rNPC and identify those who might benefit from contrast-enhanced MRI (ceMRI), patients were divided into three groups as follows: all patients underwent evaluation using the T1_T2 model initially, those with predicted values above the first-level cutoff value (internal: 0.490, external: 0.460) according to the Youden index were categorized as positive. A second-level cutoff value was established by ensuring that the sensitivity was no less than 90% across the entire test set, achieved by adding the T1WIC model to the non-positive group. Non-positive patients were then allocated to suspicious and negative groups based on the secondary cutoff values (internal: 0.197, external: 0.282) (Supplementary Figure 1). DARNDEST was optimized for layered patient management; herein, patients in the positive and suspicious groups were subjected to enhanced MR examination, whereas patients in the negative group merely underwent unenhanced MR examination, thus maximizing the cost-benefit ratio.

### Comparison of identification performance in local rNPC among models and statistical analysis

The developed models’ performance in detecting local rNPC was assessed using standard evaluation metrics such as accuracy, sensitivity, specificity, positive predictive value (PPV), and negative predictive value (NPV). And these metrics were compared between any two models using McNemar’s Z test. Receiver operating characteristic (ROC) curves were used to calculate the area under the curve (AUC) to assess the diagnostic efficacy of each model. Differences in AUCs among models were assessed using paired-sample area differences under the ROC curves. All analyses were performed using the Statistical Program for Social Sciences 22.0 (Chicago, USA). The power of the test during comparison among any two models was assessed using Power Analysis and Sample Size 2023 (Kaysville, USA).

### Cost analysis

An economic analysis was conducted based on the actual prevalence of the internal and external cohorts, positive yield of local rNPC, and associated confidence interval with a hypothetical cohort of 1,000 NPC patients. The cost of detecting one true-positive local rNPC either using enhanced or unenhanced MRI was calculated for different groups according to DARNDEST. The Chinese currency was converted to US dollars based on the exchange rate and date (rate of US$1.00=￥7.17, 2024). The prevalence was defined as those patients with local rNPC divided by the total cases. Additional fees for other examinations or treatment were not included in the economic analysis.

## Results

### Demographic and clinical characteristics of the eligible participants

Among the 1993 patients with recurrence in the internal cohort, 1467 (73.61%) and 35 (1.76%) patients had histopathologically confirmed recurrent lesion at nasopharynx and metastatic lymphadenopathy, respectively. 73.40% (138/188) and 69.11% (85/123) of patients had histopathologically confirmed local rNPC in FPHF and ACHGMU. Notably, Submucosal local rNPC accounted for 85.6%, 4.26%, and 9.76%, while lesions with necrotic characteristic accounted for 23.53%, 13.30%, and 21.95% in SYSUCC, FPHF, and ACHGMU cohorts, respectively. Most patients in the three institutions were diagnosed with advanced local rNPC, with 74.86%, 84.04%, and 82.11% in SYSUCC, FPHF, and ACHGMU, respectively. Detailed demographic and clinical information from the three institutions are listed in Supplementary Tables 3-5.

### Comparison of diagnostic performance of different models in identifying local rNPC

The T1WIC model using DenseNet showed no significant differences in overall accuracy, sensitivity, or specificity when compared to the T1WI, T2WI, or T1_T2 models, in both the internal and external cohorts (all *P* > 0.0167), with an overall accuracy and sensitivity over 83% in the internal cohort and over 81% in the external cohort, and all of the power of the test more than 95%. Radiologists achieved a slightly higher or non-inferior overall accuracy, benefiting from the favorable specificity that was slightly above or equal to that of the DenseNet models, but they experienced a decline in sensitivity, which was critical in detecting recurrence during post-treatment surveillance. Moreover, the PPV and NPV of the T1_T2 model were slightly higher than those of T1WIC without statistical significance in both test sets (Table 1). The T1_T2 model showed similar performance to the T1WIC model when using the ResNet frame, with slightly higher sensitivity but no statistical significance in both the internal (84.26% vs. 82.74%) and external (84.95% vs. 84.95%) test sets (Supplementary Table 6).

**Table 1:** Comparison of performance in identifying local rNPC among DenseNet models developed using different MRI sequence and doctors in test set

**Test**	**No of rNPC**	**TP**	**FN**	**FP**	**TN**	**sensitivity**	**specificity**	**accuracy**	**PPV**	**NPV**
						**% 95% CI (%)**	**% 95% CI (%)**	**% 95% CI (%)**	**% 95% CI (%)**	**% 95% CI (%)**
**Internal**	197									
T1WIC		166	31	35	201	84.26(79.18,89.35)	85.17(80.64,89.70)	84.76(81.37,88.14)	82.59(77.34,87.83)	86.64(82.26,91.02)
T1WI		168	29	40	196	85.28(80.33,90.23)	82.57(78.26,87.84)	84.06(80.62,87.51)	80.77(75.41,86.13)	87.11(82.73,91.49)
T2WI		164	33	40	196	83.25(78.03,88.46)	85.32(78.26,87.84)	83.14(79.61,86.67)	80.39(74.94,85.84)	85.59(81.04,90.14)
T1_T2		166	31	34	202	84.26(79.18,89.35)	85.59(81.11,90.07)	84.99(81.62,88.35)	83.00(77.79,88.21)	86.70(82.33,91.06)
Doctor-S		154	43	13	223	78.17(71.62,83.60)	94.49(90.55,96.91)	87.07(83.45,90.01)	92.22(86.78,95.62)	83.83(78.73,87.94)
Doctor-J		166	31	41	195	84.26(78.25,88.90)	82.63(77.05,87.11)	83.37(79.45,86.69)	80.19(73.97,85.26)	86.28(80.94,90.35)
**External**	93									
T1WIC		78	15	19	90	83.87(76.40,91.35)	82.57(75.45,89.69)	83.17(78.01,88.33)	80.41(72.51,88.31)	85.71(79.02,92.41)
T1WI		75	18	19	90	80.65(72.62,88.67)	82.57(75.45,89.69)	81.68(76.35,87.02)	79.79(71.67,87.91)	83.33(76.30,90.36)
T2WI		76	17	16	93	81.72(73.87,89.58)	85.32(78.68,91.96)	83.66(78.57,88.76)	82.61(74.86,90.35)	84.55(77.79,91.30)
T1_T2		79	14	18	91	84.95(77.68,92.21)	83.49(76.52,90.46)	84.16(79.12,89.19)	81.44(73.71,89.18)	86.67(80.16,93.17)
Doctor-S		77	16	3	106	82.80(73.26,89.55)	97.25(91.57,99.29)	90.59(85.49,94.09)	96.25(88.68,99.03)	86.89(79.28,92.09)
Doctor-J		72	21	4	105	77.42(67.35,85.18)	96.33(90.32,98.82)	87.62(82.09,91.68)	94.74(86.36,98.30)	83.33(75.42,89.16)

*Abbreviation*: *rNPC* recurrent nasopharyngeal carcinoma, *PPV* positive predictive value, *NPV* negative predictive value, *T1WI* T1-weighted image, *T2WI* T2-weighted image, *T1WIC* T1-weighted image post-enhancement, *Doctor-S* senior radiologist, *Doctor-J* junior radiologist.

**Fig. 2:**
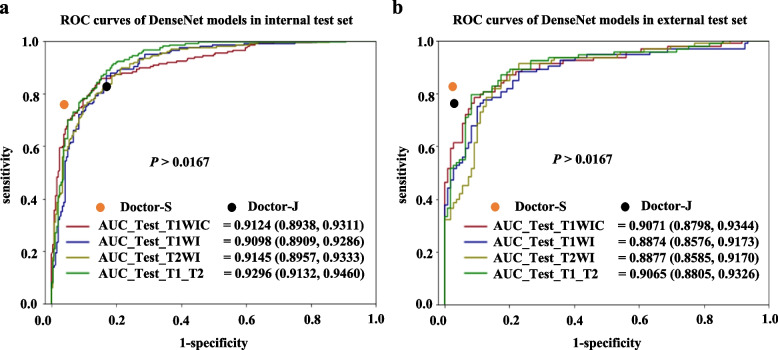
ROC curves of DenseNet models in the test set. (**a**-**b**) The diagnostic efficacy of DenseNet models developed using different MRI sequences in detecting local recurrent nasopharyngeal carcinoma is compared using ROC curves in the internal (**a**) and external (**b**) test sets. The area under the curve and 95% confidence interval of each model are also shown. Abbreviations: ROC, receiver operator characteristic curve; Doctor-S, senior radiologist; Doctor-J, junior radiologist.

Particularly, the ROC curves revealed no significant differences in the AUC between the T1WIC model and the T1WI, T2WI, or T1_T2 models in both test sets when using DenseNet (0.9124 vs. 0.9098, 0.9145, 0.9296 in the internal test set; 0.9071 vs. 0.8874, 0.8877, 0.9065 in the external test set; all *P* > 0.0167, Fig. 2) or ResNet (0.8970 vs. 0.8840, 0.8939, 0.9045 in the internal test set; 0.9061 vs. 0.8800, 0.8791, 0.8973 in the external test set; all *P* > 0.0167, Supplementary Figure 2).

### Comparison of errors in diagnosing local rNPC among models and radiologist

**Fig. 3:**
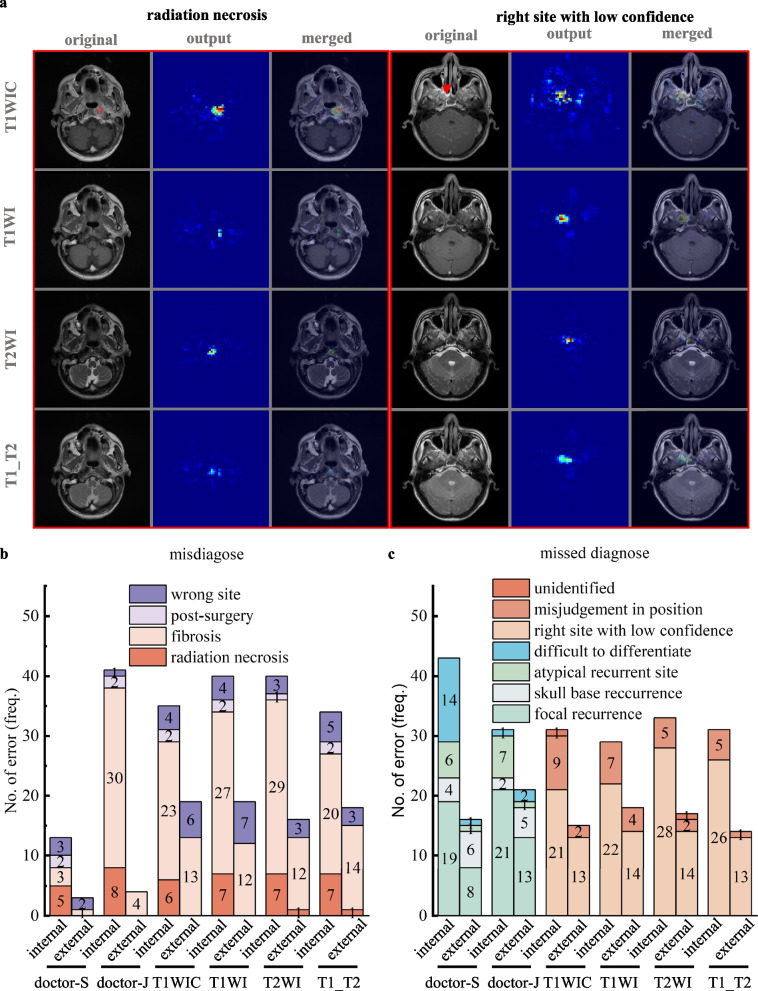
Classification and comparison of errors in diagnoses among deep learning models and doctors. **a** Representative images of major errors in diagnosing local rNPC. The representative cases are radiation necrosis (necrotic lesion indicated by asterisk) wrongly identified by models in a case without recurrence, and missed diagnose due to low confidence (early recurrent lesion indicated by arrowhead at the right parietal wall of the nasopharynx). The columns from left to right in each case are original MR images, heatmap predicted by the DenseNet model and merged images of the former two. **b**-**c** The errors of misdiagnose (**b**) and missed diagnose (**c**) in local rNPC are shown in the stacked bar chart. Herein, focal recurrence refers to cases with rT1 stage disease; atypical recurrent site refers to uncommon site involved by NPC, such as nasolacrimal duct, nasal cavity, etc.; difficult to differentiate refers to misdiagnose between local rNPC and radiation necrosis.

Further analysis of errors in diagnosing local rNPC was performed to understand the mechanism of DenseNet models in detecting lesions. In misdiagnosed cases, mistaking radiation-induced fibrosis as recurrence was the first major cause for both the DenseNet models and the junior radiologist in both test sets, whereas mistaking radiation necrosis or wrongly identifying sinusitis or enhanced turbinate as recurrence were the second major causes for the DenseNet models in the internal or external test set, respectively (Fig. 3a, b). However, the senior radiologist, whose specificity was higher than that of deep learning models and junior radiologist (94.49% vs. 82.57-85.17% in internal test set, 97.25% vs. 82.57-96.22% in external test set, Table 1), excelled in distinguishing radiation-induced fibrosis from recurrence in both test sets but encountered challenges in differentiating recurrence from radiation necrosis, leading to lower sensitivity compared to models and junior radiologist in the internal test set (78.17% vs. 83.25-85.28%, Table 1 and Fig. 3b, c).

Missed diagnoses for radiologists in both test sets were primarily due to recurrence at an early stage with focal lesions. Conversely, deep learning models struggled with accurate identification of lesions in the correct location and displayed uncertainty in true recurrent cases, with positional misjudgments as a secondary cause for missed diagnoses. Additionally, recurrence at an atypical site or skull base constituted a critical cause of missed diagnoses for radiologists in the internal or external test set, respectively (Fig. 3a, c).

### Evaluation of DARNDEST's diagnostic performance and a comparative analysis of economic and time factors against the T1_T2 model and conventional methods for detecting rNPC

Expectedly, DARNDEST showed higher accuracy and sensitivity compared to the T1_T2 model in the whole test sets, with slightly reduced specificity in both the internal (accuracy, 85.91% vs. 84.99%; sensitivity, 90.36% vs. 84.26%; specificity, 82.20% vs. 85.59%) and external (accuracy, 86.14% vs. 84.16%; sensitivity, 90.32% vs. 84.95%; specificity, 82.57% vs. 83.49%) cohorts (Table 2).

**Table 2:** Comparison of the diagnostic efficacy, total cost and time in a single MR examination using DARNDEST and T1_T2 model or conventional methods

**Cohort**	**Model/Method**	Accuracy (%)	Sensitivity (%)	Specificity (%)	TC	TT(hr)
		95% CI (%)	95% CI (%)	95% CI (%)		
**Internal**	**T1_T2**	84.99 (81.62,88.35)	84.26 (79.18,89.35)	85.59 (81.11,90.07)	￥2,246,000($313,250)	129.2
	**DARNDEST**	85.91 (82.19,88.98)	90.36 (85.13,93.94)	82.20 (76.59,86.74)	￥2,371,293($330,724)	190.7
**External**	**T1_T2**	84.16 (79.12,89.19)	84.95 (77.68,92.21)	83.49 (76.52,90.46)	￥2,246,000($313,250)	129.2
	**DARNDEST**	86.14 (80.42,90.44)	90.32 (81.97,95.20)	82.57 (73.86,88.92)	￥2,358,723($328,971)	184.5
**Internal/External**	**Conventional**	/	/	/	￥2,444,000($340,865)	226.4

*Abbreviation*: *TC* total cost, *TT* Total time, *DARNDEST* deep-learning-assisted recurrent NPC detecting simultaneous tactic.

**Fig 4:**
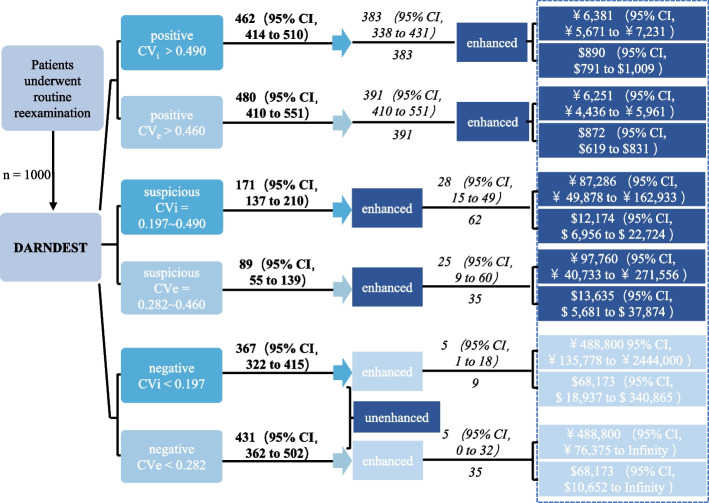
Comparison of the economic burden associated with using enhanced MRI in each group based on DARNDEST. The economic analysis was modeled based on the actual prevalence, positive yield of local rNPC, and associated CI derived from this study with a hypothetical cohort of 1,000 patients who underwent follow-up MR examination after radical treatment. For each group in the figure, numbers in bonds denote cases and associated CI categorized as positive, suspicious or negative by DARNDEST. Italicized numbers above the line represent true-positive cases of local rNPC detected by DARNDEST, while those below represent true-positive cases confirmed by histopathology or follow-up MRI. Dark blue text boxes labeled "enhanced/unenhanced" is recommended MR examination methods according to DARNDEST. Data in gridline represent the costs per true-positive case of each group. Cost in China from the Medical Insurance Administration Bureau of Guangzhou, 2024. One dollar = 7.17 RMB. Abbreviation: CV_i_ or CV_e_, cut-off value of the internal or external set, respectively

Further economic analysis was compared among the conventional method, T1_T2 model alone and DARNDEST. There would be 462, 171 and 367 patients categorized as positive, suspicious and negative group in the internal cohort according to DARNDEST, of which 383, 62 and 9 patients were diagnosed with local rNPC based on the actual incidence of the internal cohort with the hypothesis of 1000 patients. Meanwhile, 383, 28 and 5 patients would be detected as having local rNPC precisely using DARNDEST. ￥6,381 (5,671, 7,231), ￥87,286 (49,878, 162,933) and ￥488,800 (135,778, 244,000) were needed to detect a patient with local rNPC in each group. Similarly, there would be 480, 89 and 431 patients categorized as positive, suspicious and negative group, of which 391, 35 and 35 patients were diagnosed with local rNPC based on the actual incidence of external cohort, while 391, 25 and 5 patients would be detected as local recurrence using DARNDEST. ￥6,251 (4,436, 5,961), ￥97,760 (40,733, 271,556) and ￥488,800 (76,375, infinity) were needed to detect a patient with recurrence in each group (Fig. 4 and Supplementary Table 7).

For a single examination in a hypothetical cohort of 1,000 patients, DARNDEST required 190.7 and 184.5 hours in the internal and external cohorts, while the T1_T2 model and conventional method required 129.2 and 226.4 hours, respectively. The total cost of MR examination using DARNDEST was 105.58% and 100.53% of the cost of the T1_T2 model (￥2,371,293 vs ￥2,246,000 and ￥2,358,723 vs ￥2,246,000, respectively) in the internal and external cohorts, whereas it was 97.03% and 96.51% (￥2,371,293 vs ￥2,444,000 and ￥2,358,723 vs ￥2,444,000, respectively) of the cost of the conventional method in the internal and external cohorts (Table 2).

## Discussion

Our study showcased the viability of unenhanced MRI for detecting local rNPC during post-treatment surveillance using widely adopted deep learning frameworks, DenseNet or ResNet, validated across internal and external cohorts. The combined T1WI and T2WI model exhibited sensitivity comparable to that of radiologists. Error analysis revealed analogous misdiagnosis and missed diagnosis patterns for DenseNet models and radiologists, indicating similar lesion detection mechanisms. Additionally, we introduced an efficient and economical follow-up strategy using DenseNet, DARNDEST, optimizing sensitivity by overlaying the T1WIC model onto the T1_T2 model in positive and suspicious subpopulations while maintaining cost-effectiveness.

Early detection of recurrent NPC is crucial for timely salvage treatment [4]. Routine annual MR examination of the nasopharynx and neck is essential for NPC surveillance since submucosal or intracranial lesions can be missed during physical examination or endoscopy [4, 8, 22]. Detecting submucosal recurrent lesions and early-stage disease is challenging, and distinguishing recurrent NPC from radiation-induced alterations is complex [4, 12]. Conventional MRI has limited sensitivity and specificity, with 56% sensitivity and 78-83% specificity reported since it only relied on the morphological characteristics [10]. However, our DenseNet models achieved higher sensitivity (83.25-84.26% internal, 80.65-84.95% external) and comparable specificity (82.57-85.59% internal, 82.57-85.32% external). Moreover, the optimized DARNDEST gained an impressively increased sensitivity (internal, 90.36% and external, 90.32%) with mild reduction in specificity (internal, 82.20% and external, 82.57%); still comparable to previous studies and could be supplemented by radiologists.

Additionally, although several studies have demonstrated a lack of added clinical value of using gadolinium contrast agent for routine follow-up scans of benign intracranial tumors (e.g., meningiomas [23], pituitary macroadenoma [24] and vestibular schwannomas [25]), cystic lesions of pancreas [26, 27] and small solid renal masses [28], we have similarly shown that enhanced MRI can be exempted in the initial detection of NPC with the aid of a deep learning model [17]. However, no studies have evaluated unenhanced MRI or abbreviated MRI protocols for imaging follow-up of NPC, nor have they addressed the economic impact of eliminating gadolinium contrast agent. Herein, the economic analysis revealed that using DARNDEST could save significant costs (13.4/15.6 and 76.6/78.2 times) in detecting positive cases of local rNPC in the suspicious or negative groups compared to conventional methods. Moreover, DARNDEST reduced MR examination costs and time by approximately ￥72,707/85,277 ($10,140/11,894) and 35.7/41.9 hours in a hypothetic cohort of 1,000 patients based on the actual incidence of local rNPC in the internal or external cohorts, respectively. Taken together, this demonstrated for the first time in the era of artificial intelligence, enhanced MR examination may be exempt reasonably in the administration of NPC patients after radical treatment, since not all patients are indicated for gadolinium contrast agents, especially in those patients with impaired renal function induced by multiple causes, including chemotherapy [29–31]. Particularly, eliminating gadolinium contrast agent for the follow-up of NPC could lead to significant cost and time savings, while improving accessibility and patient tolerance.

Notably, the T1_T2 model exhibited slightly higher or equal sensitivity but lower specificity compared to the radiologists, indicating that unenhanced MRI was sufficient for detecting local lesions in unsupervised situations, whereas enhanced MRI aided in differentiation for radiologists. Particularly, the T1_T2 model performed consistently in both cohorts, while radiologists showed variations, likely due to distinct disease characteristics and scanning parameters between institutions; for example, the proportion of rNPC cases exhibiting necrotic characteristics was higher in the internal cohort than in the external cohort and the postenhancement axial T1-weighted images of the external cohort were fat saturated, different from those of the internal cohort. Obviously, variable data resources have helped to improve model robustness. Moreover, causes of error in misdiagnoses or missed diagnoses among DenseNet models developed using either unenhanced or enhanced MRI were consistent. Specifically, the models tended to misconstrued the asymmetric structure with a mass effect as a recurrent lesion, such as fibrotic scarred tissue induced by radiation, and were prone to miss diagnosis in cases with limited lesions although localized lesion precisely but insufficient confidence, similar to that of junior radiologist. Therefore, we speculated that shape or texture might play a more important role than intensity in the recognition of deep convolution neural networks (DCNNs) in grayscale images. Consistent with previous studies, DCNNs have access to local shape information in the form of local edge relations in object classification [32, 33]. However, further comparative studies focusing on feature extraction in identification using DCNNs are warranted in the future.

The study has some limitations. First, a portion of patients (internal, 24.64%; external, 25.53% and 30.89%) were diagnosed based on time-serial MRI without histopathological confirmation due to challenging biopsy locations. However, debatable cases were excluded initially, and the remaining enrolled cases were confirmed by either detecting size enlargement through follow-up MRIs or observing lesion shrinkage after anti-cancer treatment. Second, the focus was on detecting local rNPC for overall accuracy when developing deep learning models, omitting considerations for metastatic lymph nodes or tumor segmentation. Third, only single-time point axial images were used for model development since the purpose of this study was to determine the feasibility of detecting local rNPC using unenhanced MRI. Abbreviated protocols should also be explored for follow-up patients in the era of artificial intelligence, where time-serial images and multiple orientation images should be considered in future studies. On the other hand, time-serial MRI examinations during NPC follow-up could provide additional insights in clinical practice, whereas a single-time point MRI examination may lead to an underestimation of the radiologists' performance in this study. Fourth, clinical data, particularly Epstein-Barr Virus DNA copy numbers, were not incorporated into the models. Future studies should consider comprehensive models that integrate clinical data and time-serial images. Lastly, for cases that might benefit from enhanced MRI, a repetition of the MRI scan with contrast agent could introduce additional costs, which may outweigh the initial savings from eliminating the contrast agent. Therefore, the developed T1_T2 models should be integrated into the scanning system to provide real-time guidance during follow-up scans.

## Conclusion

Using unenhanced MRI with deep learning models for detecting local rNPC is feasible. DARNDEST's layer management during follow-up is efficient, economic, timesaving, and minimally toxic.

## Supplementary Information

Additional file 1.

Additional file 2.

## Data Availability

De-identified data supporting this study may be shared based on reasonable written request to the corresponding author. Access to de-identified data will require a Data Access Agreement and IRB clearance, which will be considered by the institutions who provided the data for this research.

## Abbreviations

AUC: area under curve
ceMRI: contrast-enhanced magnetic resource images
DARNDEST: deep-learning-assisted recurrent NPC detecting simultaneous tactic
DICOM: digital imaging and communications in medicine
DCNNs: deep convolution neural networks
NPV: negative predictive value
PPV: positive predictive value
rNPC: recurrent nasopharyngeal carcinoma
ROC: receiver operating characteristic curve
T1WI: T1-weighted image
T2WI: T2-weighted image
T1WIC: post-contrast T1WI
