# Supplementary material for: Establishment of a deep-learning-assisted recurrent nasopharyngeal carcinoma detecting simultaneous tactic (DARNDEST) with high cost-effectiveness based on magnetic resonance images: a multicenter study in an endemic area
Source: Cancer Imaging. 2025 Mar 24;25:39. doi: 10.1186/s40644-025-00853-5 (PMC11931764; doi:10.1186/s40644-025-00853-5)
Supplement: Supplementary file 1 — Additional file 1. [file 40644_2025_853_MOESM1_ESM.pdf]

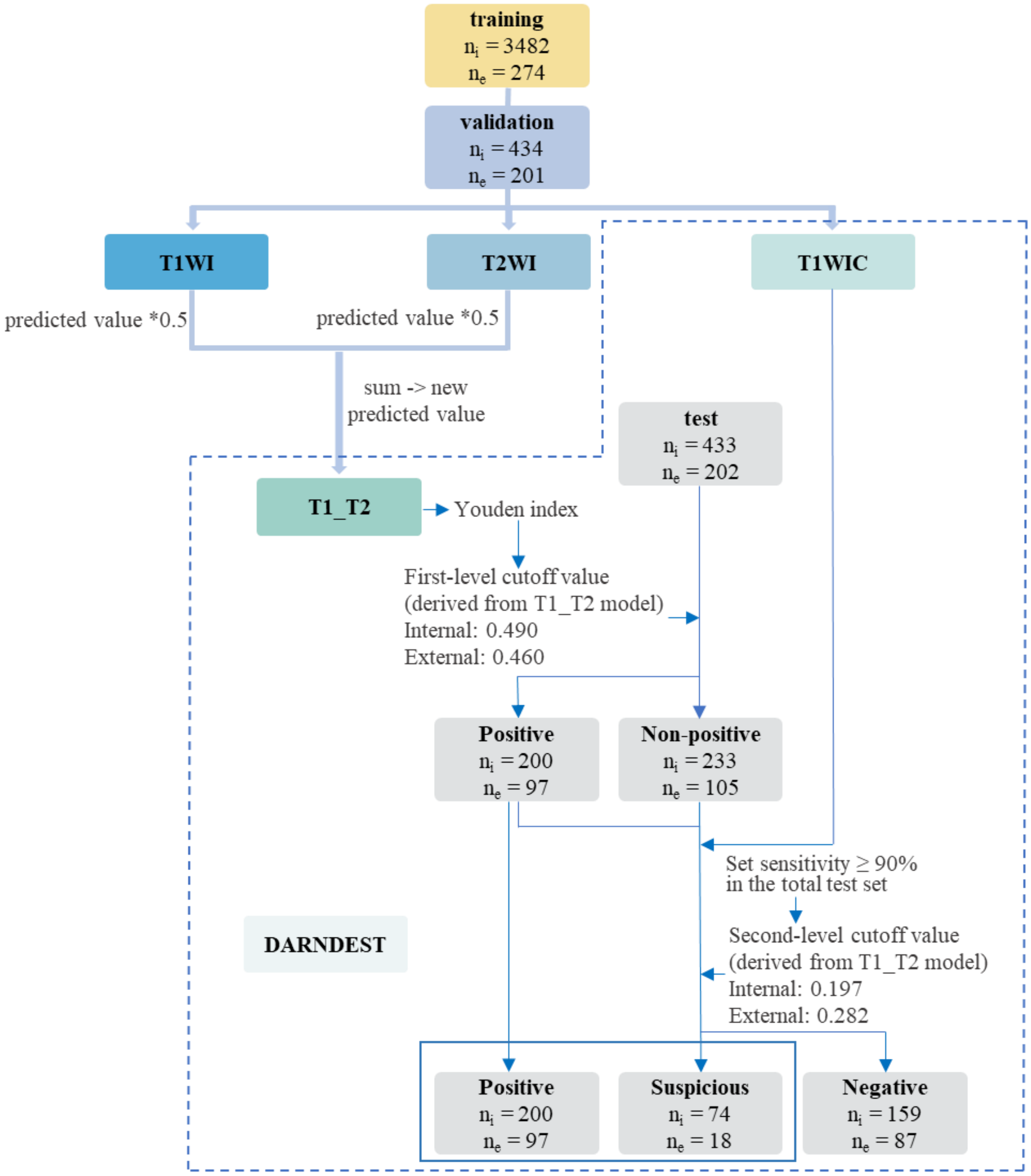

Supplementary Figure 1 Optimization and workflow of the DARNDEST

The optimization and workflow of DARNDEST was showed in blue gridline. Specially, patients underwent evaluation using the T1_T2 model initially, those with predicted values above the first-level cutoff value (internal: 0.490, external: 0.460) according to the Youden index were categorized as positive. A secondary cutoff value was established by ensuring that the sensitivity was no less than 90% across the entire test set, achieved by adding the T1WIC model to the non-positive group. Non-positive patients were then allocated to suspicious and negative groups based on the secondary cutoff values (internal: 0.197, external: 0.282). The positive and suspicious groups, highlighted in a blue solid frame, were recommended to undergo enhanced MRI, while the negative group was advised to use unenhanced MRI during follow-up, according to DARNDEST.

Abbreviation: DARNDEST, deep-learning-assisted recurrent NPC detecting simultaneous tactic.

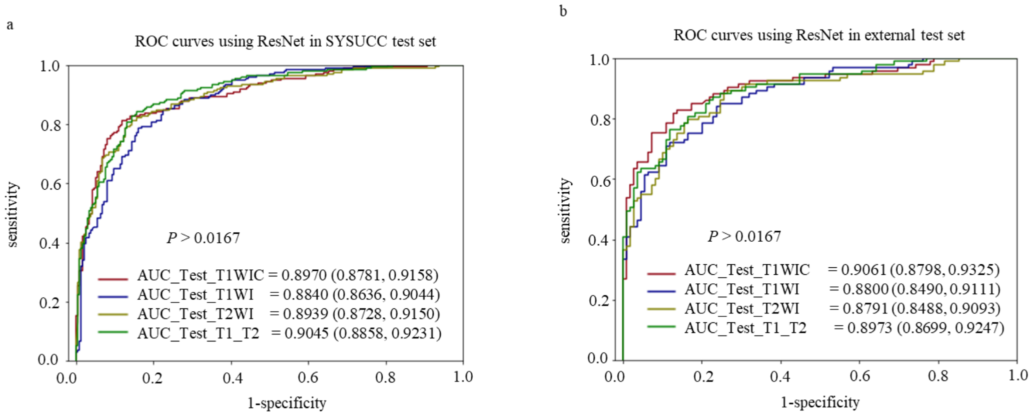

Supplementary Figure 2 ROC curves of ResNet models in test set

(a-b) The diagnostic efficacy in detecting local recurrent nasopharyngeal carcinoma of ResNet models developed using different MRI sequences were compared using ROC curves in internal (a) and external (b) test set. Area under curve and 95% confidence interval of each model was also shown. Abbreviation: ROC, receiver operator characteristic curve.
