# Supplementary material for: Establishment of a deep-learning-assisted recurrent nasopharyngeal carcinoma detecting simultaneous tactic (DARNDEST) with high cost-effectiveness based on magnetic resonance images: a multicenter study in an endemic area
Source: Cancer Imaging. 2025 Mar 24;25:39. doi: 10.1186/s40644-025-00853-5 (PMC11931764; doi:10.1186/s40644-025-00853-5)
Supplement: Supplementary file 2 — Additional file 2. [file 40644_2025_853_MOESM2_ESM.pdf]

Supplementary Methods

The preprocessing of MR images

The input 3D image consists of 16 slices, covering the structures of the nasopharynx. To meet the input requirements of the model, all MR images were sampled using bilinear interpolation to a size of 384*384 pixels. Before being fed into the model, each images undergo a standardization process using the following steps:

(1) Calculate the pixel mean value "u" of the images

$$u = \frac{1}{N}\sum_{k=0}^{N} X_k$$

X represents the set of pixels in the image, *N* represents the total number of pixels in the image, and X_k represents the value of the ith pixel.

(2) Calculate the pixel mean value "m" of the foreground of the images

$$m = \frac{1}{\sum_k^N 1_{(X_k>u)}}\sum_{k=0}^{N} X_{k_{(X_k>u)}}$$

u represents the average value of pixels in the image.

(3) Obtain the standardized images "Z"

$$Z = \frac{X}{m}$$

m represents the average value of pixels in the foreground of the image.

The structure of the model

The 3D DenseNet model used an end-to-end two-class tasking learning network architecture. In order to obtain rich and representative features, the 3D Densenet has 6 Denseblock and 5 Transitionblock. Each Denseblock contained several densely connected convolutional layers, and each convolutional layer (conv) was followed by a Batch Normalization layer (BN) and the Leaky Rectified Linear Unit activation (LeakyReLU). DenseNet employs 1×1×1 convolution as bottleneck layer in DenseBlock structure [BN+LeakyReLU+conv1×1×1+BN+LeakyReLU + conv3×3×3]. Transitionblock consisted of a convolutional layer and a pooling layer. The Transition Block typically consists of multiple layers, including an activation layer, a Batch Normalization layer, a convolutional layer, and a pooling layer. The structure is defined as [BN+LeakyReLU+conv1×1×1+maxpool]. The model takes the nasopharynx 3D MR image as input and provides the diagnostic probability as output.

Supplementary Table 1 Detailed parameters of 3D DenseNet models

Layers	Output Size	3D DenseNet
Convolution	16 x 192 x 192	3 x 3 x 3 conv, stride 1 x 2 x 2
DenseBlock (1)	16 x 192 x 192	[1 x 1 x 1 conv<br>3 x 3 x 3 conv] x 3
TransitionBlock (1)	16x 192 x 192	1 x 1 x 1 conv
	16 x 96 x 96	1 x 1 x 1 max pool, stride 1 x 2 x 2
DenseBlock (2)	16 x 96 x 96	[1 x 1 x 1 conv<br>3 x 3 x 3 conv] x 4
TransitionBlock (2)	16 x 96 x 96	1 x 1 x 1 conv
	8 x 48 x 48	1 x 1 x 1 max pool, stride 2 x 2 x 2
DenseBlock (3)	8 x 48 x 48	[1 x 1 x 1 conv<br>3 x 3 x 3 conv] x 5
TransitionBlock (3)	8 x 48 x 48	1 x 1 x 1 conv
	8 x 24 x 24	1 x 1 x 1 max pool, stride 1 x 2 x 2
DenseBlock (4)	8 x 24 x 24	[1 x 1 x 1 conv<br>3 x 3 x 3 conv] x 6
TransitionBlock (4)	8 x 24 x 24	1 x 1 x 1 conv
	4 x 12 x 12	1 x 1 x 1 max pool, stride 2 x 2 x 2
DenseBlock (5)	4 x 12 x 12	[1 x 1 x 1 conv<br>3 x 3 x 3 conv] x 7
TransitionBlock (5)	4 x 12 x 12	1 x 1 x 1 conv
	4 x 6 x 6	1 x 1 x 1 max pool, stride 1 x 2 x 2
DenseBlock (6)	4 x 6 x 6	[1 x 1 x 1 conv<br>3 x 3 x 3 conv] x 9
ClassificationLayer	1 x 1 x 1	4 x 6 x 6 global average pool
	256	256D fully-connected
	1	1D fully-connected, sigmoid

Supplementary Table 2 Cutoff values of predicted values of different models.

Model	T1WIC	T1WI	T2WI	T1_T2
**Internal corhot**				
DenseNet	0.429	0.500	0.520	0.490
ResNet	0.381	0.350	0.624	0.475
**External corhot**				
DenseNet	0.417	0.408	0.477	0.460
ResNet	0.376	0.362	0.315	0.380

Supplementary Table 3 Demographic and clinical characteristics of the eligible participants in different sets of internal cohort (SYSUCC).

Characteristics	All<br>No. (%)	Training set<br>No. (%)	Validation set<br>No. (%)	Test set<br>No. (%)
**No. of participants**	4349	3482	434	433
**Age (y)**	46.8±10.61	46.8±10.67	47.1 ± 10.44	46.6 ± 10.28
**Gender**				
Male	3222 (74.09)	2575 (73.95)	328 (75.58)	319 (73.67)
Female	1127 (25.91)	907 (26.05)	106 (24.42)	114 (26.33)
**Histopathology of rNPC**				
local rNPC	1467 (73.61)	1175 (73.76)	150 (73.89)	142 (72.08)
lymph node metastasis	35 (1.76)	29 (1.82)	2 (0.99)	4 (2.03)
Clinical diagnosis[a]	491 (24.64)	389 (24.42)	51 (25.12)	51 (25.89)
**Imaging Feature of rNPC**				
submucosal	1706 (85.60)	1368 (85.88)	167(82.27)	171 (86.80)
necrotic	469 (23.53)	371 (23.29)	48 (23.65)	50 (25.38)
**Histopathology of post-therapy**				
inflammation	75 (3.18)	61(3.23)	6(2.60)	8(3.39)
Clinical diagnosis[b]	2281 (96.82)	1828 (96.77)	225 (97.40)	228 (96.61)
**rT stage of rNPC**				
rT1	218 (10.94)	173 (10.80)	18 (8.87)	27 (13.71)
rT2	283 (14.20)	223 (14.00)	36 (17.73)	24 (12.18)
rT3	1014 (50.88)	834 (52.35)	89 (43.84)	91 (46.19)
rT4	478 (23.98)	363 (22.79)	60 (29.56)	55 (27.92)

[a]The subpopulation of participants were diagnosed as local recurrence NPC according to MRI without confirmation of histopathology.

[b]The subpopulation of participants were diagnosed as post-treatment change without local recurrence according to MRI, no evidence of malignancies at nasopharynx has been observed after followed up over one year for these participants.

Abbreviation: NPC, nasopharyngeal carcinoma; SYSUCC, Sun Yat-sen University Cancer Center.

Supplementary Table 4 Demographic and clinical characteristics of the eligible participants in different sets of external cohort I (FPHF).

Characteristics	All<br>No. (%)	Training set<br>No. (%)	Validation set<br>No. (%)	Test set<br>No. (%)
**No. of participants**	420	169	125	126
**Age (y)**	51.8 ±11.4	52.1 ± 10.6	51.6 ± 12.2	51.8±11.5
**Gender**				
Male	289 (68.81)	110 (65.09)	90 (72.00)	89 (70.63)
Female	131 (31.19)	59 (34.91)	35 (28.00)	37 (29.37)
**Histopathology of rNPC**				
local rNPC	138 (73.40)	58 (76.32)	40 (71.43)	40 (71.43)
lymph node metastasis	2 (1.06)	1 (1.32)	0 (0.00)	1 (1.79)
Clinical diagnosis[a]	48 (25.53)	17 (22.37)	16 (28.57)	15 (26.79)
**Imaging Feature of rNPC**				
submucosal	8 (4.26)	3 (3.95)	1 (1.79)	4 (7.14)
necrotic	25 (13.30)	11 (14.47)	8 (14.29)	6 (10.71)
**Histopathology of post-therapy**				
Clinical diagnosis[b]	232 (100.00)	93 (100.00)	69 (100.00)	70 (100.00)
**rT stage of NPC**				
rT1	10 (5.32)	4 (5.26)	2 (3.57)	4 (7.14)
rT2	20 (10.64)	12 (15.79)	6 (10.71)	2 (3.57)
rT3	65 (34.57)	22 (28.95)	21 (37.50)	22 (39.29)
rT4	93 (49.47)	38 (50.00)	27 (48.21)	28 (50.00)

[a]The subpopulation of participants were diagnosed as local recurrence NPC according to MRI without confirmation of histopathology.

[b]The subpopulation of participants were diagnosed as post-treatment change without local recurrence according to MRI, no evidence of malignancies at nasopharynx has been observed after followed up over one year for these participants.

Abbreviation: NPC, nasopharyngeal carcinoma; FPHF, First People's Hospital of Foshan.

Supplementary Table 5 Demographic and clinical characteristics of the eligible participants in different sets of external cohort II (ACHGMU).

**Characteristics**	**All** No. (%)	**Training set** No. (%)	**Validation set** No. (%)	**Test set** No. (%)
**No. of participants**	257	105	76	76
**Age (y)**	49.4 ±11.2	51.1 ±10.7	47.2 ±10.7	49.1 ±11.5
**Gender**				
Male	166 (64.59)	74 (70.48)	42 (55.26)	50 (65.79)
Female	91 (35.41)	31 (29.52)	34 (44.74)	26 (34.21)
**Histopathology of rNPC**				
local rNPC	85 (69.11)	36 (73.47)	26 (70.27)	23 (62.16)
Clinical diagnosis[a]	38 (30.89)	13 (26.53)	11 (29.73)	14 (37.84)
**Imaging Feature of rNPC**				
submucosal	12 (9.76)	6 (12.25)	3 (8.11)	3 (8.11)
necrotic	27 (21.95)	9 (18.37)	7 (18.92)	11 (29.73)
**Histopathology of post-therapy**				
Clinical diagnosis[b]	134 (100.00)	56 (100.00)	39 (100.00)	39 (100.00)
**rT stage of NPC**				
rT1	6 (4.88)	0 (0.00)	4 (10.81)	2 (5.41)
rT2	16 (13.01)	9 (18.37)	2 (5.41)	5 (13.51)
rT3	31 (25.20)	10 (20.41)	12 (32.43)	9 (24.32)
rT4	70 (56.91)	30 (61.22)	19 (51.35)	21 (56.76)

[a]The subpopulation of participants were diagnosed as local recurrence NPC according to MRI without confirmation of histopathology.

[b]The subpopulation of participants were diagnosed as post-treatment change without local recurrence according to MRI, no evidence of malignancies at nasopharynx has been observed after followed up over one year for these participants.

Abbreviation: NPC, nasopharyngeal carcinoma; ACHGMU, Affiliated Cancer Hospital of Guangzhou Medical University.

Supplementary Table 6 Comparison of performance in identifying local rNPC among ResNet models developed using different MRI sequence in test set.

Test	No. of rNPC	TP	FN	FP	TN	sensitivity % 95% CI (%)	specificity % 95% CI (%)	accuracy % 95% CI (%)	PPV % 95% CI (%)	NPV % 95% CI (%)
**Internal**	197									
T1WIC		163	34	39	197	82.74(77.46,88.02)	83.47(78.74,88.21)	83.14(79.61,86.67)	80.69(75.25,86.14)	85.28(80.71,89.85)
T1WI		156	41	46	190	79.19(73.52,84.86)	80.51(75.45,85.56)	79.91(76.13,83.68)	77.23(71.44,83.01)	82.25(77.32,87.18)
T2WI		162	35	38	198	82.23(76.90,87.57)	83.90(79.21,88.59)	83.14(79.61,86.67)	81.00(75.56,86.44)	84.98(80.39,89.57)
T1_T2		166	31	38	198	84.26(79.18,89.35)	83.90(79.21,88.59)	84.06(80.62,87.51)	81.37(76.03,86.72)	86.46(82.03,90.89)
**External**	93									
T1WIC		79	14	22	87	84.95(77.68,92.21)	79.82(72.28,87.35)	82.18(76.90,87.46)	78.22(70.17,86.27)	86.14(79.40,92.88)
T1WI		73	20	23	86	78.49(70.14,86.84)	78.90(71.24,86.56)	78.71(73.07,84.36)	76.04(67.50,84.58)	81.13(73.68,88.58)
T2WI		77	16	27	82	82.80(75.13,90.47)	75.23(67.13,83.33)	78.71(73.07,84.36)	74.04(65.61,82.46)	83.67(76.36,90.99)
T1_T2		79	14	23	86	84.95(77.68,92.21)	78.90(71.24,86.56)	81.68(76.35,87.02)	77.45(69.34,85.56)	86.00(79.20,92.80)

Abbreviation: PPV, positive predictive value; NPV, negative predictive value; T1WI, T1-weighted image; T2WI, T2-weighted image; T1WIC, post-contrast T1-weighted image.

Supplementary Table 7 Detailed calculation method for estimating the number of participants in each group based on DARNDEST

	Group	Cut-off value[a]	Percentage of participants (%)[b]	No. of participants[c]	Percentage of ground-true positive rNPC (%)[d]	No. of ground-true positive rNPC[e]	Percentage of leakproof rNPC by DARNDEST (%)[f]	No. of leakproof rNPC by DARNDEST[g]
**Internal**	Positive	> 0.490	46.19 (41.43, 51.01)	462 (414, 510)	38.34 (33.77, 43.12)	383 (338, 431)	/	/
	suspicious	0.197~0.490	17.09 (13.73, 21.04)	171 (137, 210)	6.24 (4.23, 9.05)	62 (42, 91)	2.77 (1.51, 4.93)	28 (15, 49)
	negative	< 0.197	36.72 (32.20, 41.48)	367 (322, 415)	0.92 (0.30, 2.51)	9 (3, 25)	0.46 (0.08, 1.84)	5 (1, 18)
**External**	positive	> 0.460	48.02 (40.99, 55.13)	480 (410, 551)	39.11 (32.41, 46.23)	391 (324, 462)	/	/
	suspicious	0.282~0.460	8.91 (5.52, 13.93)	89 (55, 139)	3.47 (1.53, 7.30)	35 (15, 73)	2.48 (0.91, 6.00)	25 (9, 60)
	negative	< 0.282	43.07(36.19, 50.21)	431 (362, 502)	3.47 (1.53, 7.30)	35 (15,73)	0.50 (0.03, 3.15)	5 (0, 32)

[a]Cut-off value of each group when adopting DARNDEST, the cut-off value was derived from T1_T2 model.
[b]The percentage of participant recognized as positive, suspicious or negative rNPC in the test set according to DARNDEST.
[c]Number of participants of each group in the hypothetical cohort of 1,000 patients when adopting DARNDEST (No. of participants in each group = percentage of participants (%) * 1000).
[d]The percentage of ground-true positive rNPC in the test set.
[e]Number of ground-true positive rNPC in the hypothetical cohort based on the actual ratio of ground-true positive rNPC in the test set.
[f]The percentage of ground-true positive rNPC missing by T1_T2 model but recognized as positive rNPC by T1WIC model in the test set.
[g]Number of ground-true positive rNPC missing by T1_T2 model but recognized as positive rNPC by T1WIC model in the hypothetical cohort based on the actual ratio of that in the test set.
Abbreviation: rNPC, recurrent nasopharyngeal carcinoma.
